# 4429 Powering precision medicine research with the efficient construction of large diverse cohorts

**DOI:** 10.1017/cts.2020.341

**Published:** 2020-07-29

**Authors:** Lynn Petukhova, Nana Ekua Adenu-Mensah, Ning Shang, Lyudmila Ena, Carol Friedman, Chunhua Weng, Eric W. Schrimshaw

**Affiliations:** 1 Columbia University, Irving Institute for Clinical; 2 Columbia University; 3 University of Central Florida College of Medicine

## Abstract

OBJECTIVES/GOALS: There is an imperative need to initiate translational genetic studies of hidradenitis suppurativa (HS). Such work requires large cohorts and no HS registries exist. Precision medicine initiatives provide new resources and methods for efficiently constructing cohorts, but empirically informed best practice guidelines are needed. METHODS/STUDY POPULATION: Traditional methods for building cohorts rely on clinical encounters to identify patients and collect phenotype data. Precision medicine initiatives aim to decrease the time and cost of data collection by using alternative sources, including electronic health records (EHR) and remote collection of patient-reported data. The public’s use of the Internet to obtain and exchange health-related information coupled with the success of direct-to-consumer genetic companies suggests that it is feasible to remotely ascertain research participants for genetic studies. Importantly, Internet cohorts provide an opportunity to include research participants who are disconnected from healthcare, and thus remain hidden from research that relies on EHR or clinical services. RESULTS/ANTICIPATED RESULTS: First, to conduct studies in EHR we are developing an analytic pipeline for the automated extraction of an accurate HS diagnosis using natural language processing of clinical notes. In our preliminary work we are also using ICD codes to build cohorts in two EHR systems with and without linked genetic data. Second, we have developed Internet advertising campaigns for symptom-based recruitment. Informed consent and patient-reported data is collected on-line through a series of short surveys. Patients who complete the surveys and express interest in participating in genetic studies are sent saliva collection kits and return mailing material. Finally, we have established an HS biobank that has DNA from 300 participants identified through clinical services. Enrollment is on going. DISCUSSION/SIGNIFICANCE OF IMPACT: Our goal is to assemble an HS cohort that is large enough to power genetic discoveries. Our work is generating empirical evidence for precision medicine guidelines and will improve our knowledge about HS. The methods we are developing can be applied to efficiently create new cohorts for genetic studies of other diseases across different clinical areas.

